# A Novel Surface Electrocardiogram–Based Marker of Ventricular Arrhythmia Risk in Patients With Ischemic Cardiomyopathy

**DOI:** 10.1161/JAHA.112.001552

**Published:** 2012-08-24

**Authors:** William B. Nicolson, Gerry P. McCann, Peter D. Brown, Alastair J. Sandilands, Peter J. Stafford, Fernando S. Schlindwein, Nilesh J. Samani, G. André Ng

**Affiliations:** Department of Cardiovascular Sciences, University of Leicester, Leicester, UK (W.B.N., P.D.B., N.J.S., G.A.N.); Department of Engineering, University of Leicester, Leicester, UK (F.S.S.); National Institute for Health Research Leicester Cardiovascular Biomedical Research Unit, Glenfield Hospital, Leicester, UK (W.B.N., G.P.M., N.J.S., G.A.N.); University Hospitals of Leicester NHS Trust, Leicester, UK (G.P.M., A.J.S., P.J.S., N.J.S., G.A.N.)

**Keywords:** sudden, death, electrical restitution, risk factors, implantable cardioverter defibrillator, electrocardiography

## Abstract

**Background:**

Better sudden cardiac death risk markers are needed in ischemic cardiomyopathy (ICM). Increased heterogeneity of electrical restitution is an important mechanism underlying the risk of ventricular arrhythmia (VA). Our aim was to develop and test a novel quantitative surface electrocardiogram–based measure of VA risk in patients with ICM: the Regional Restitution Instability Index (R2I2).

**Methods and Results:**

R2I2, the mean of the standard deviation of residuals from the mean gradient for each ECG lead at a range of diastolic intervals, was measured retrospectively from high-resolution 12-lead ECGs recorded during an electrophysiology study. Patient groups were as follows: Study group, 26 patients with ICM being assessed for implantable defibrillator; Control group, 29 patients with supraventricular tachycardia undergoing electrophysiology study; and Replication group, 40 further patients with ICM. R2I2 was significantly higher in the Study patients than in Controls (mean ± standard error of the mean: 1.09±0.06 versus 0.63±0.04, *P*<0.001). Over a median follow-up period of 23 months, 6 of 26 Study group patients had VA or death. R2I2 predicted VA or death independently of demographic factors, electrophysiology study result, left ventricular ejection fraction, or QRS duration (Cox model, *P*=0.029). R2I2 correlated with peri-infarct zone as assessed by cardiac magnetic resonance imaging (r=0.51, *P*=0.024). The findings were replicated in the Replication group: R2I2 was significantly higher in 11 of 40 Replication patients experiencing VA (1.18±0.10 versus 0.92±0.05, *P*=0.019). In combined analysis of ICM cohorts, R2I2 ≥1.03 identified subjects with significantly higher risk of VA or death (43%) compared with R2I2 <1.03 (11%) (*P*=0.004).

**Conclusions:**

In this pilot study, we have developed a novel VA risk marker, R2I2, and have shown that it correlated with a structural measure of arrhythmic risk and predicted risk of VA or death in patients with ICM. R2I2 may improve risk stratification and merits further evaluation. (***J Am Heart Assoc*. 2012;1:e001552 doi: 10.1161/JAHA.112.001552.**)

## Introduction

Sudden cardiac death (SCD) is responsible for >3 million deaths per year worldwide.^1^ Implantable cardioverter-defibrillators (ICDs) are of proven benefit in preventing SCD. Still, SCD risk assessment has considerable limitations despite 20 years of research.^2^ In the Multicenter Automatic Defibrillator Implantation Trial II (MADIT II) and Sudden Cardiac Death in Heart Failure Trial (SCD-HeFT), <40% of patients received appropriate ICD shock therapy during the first 4 years of follow-up,^3,4^ while the majority of SCD occurrs in the large population of people who, according to current evidence, are stratified as being at “low risk.”^3^

Ventricular arrhythmia (VA) risk is known to be associated with heterogeneity of repolarization.^4,5^ Increasing evidence from computer and animal models now implicates increased heterogeneity of electrical restitution as an important arrhythmogenic mechanism.^6^ Action potential duration (APD) restitution describes a myocardial property whereby APD is determined by the preceding diastolic interval. In a small canine study, body surface measurement of APD restitution has been shown to be possible with the use of a single electrocardiogram (ECG) lead and has been correlated with epicardial unipolar electrograms.^7,8^

The aim of this retrospective study was to use the surface ECG to develop a new electrophysiological measure of electrical restitution heterogeneity: the Regional Restitution Instability Index (R2I2). We sought to assess its correlation with cardiac magnetic resonance (CMR) features of increased VA risk and to test and replicate its utility in predicting risk of VA or death in patients with ischemic cardiomyopathy (ICM) who were candidates for ICD therapy.^9–11^

## Methods

### Subjects

Ethical approval for this study was sought but deemed unnecessary by the Leicestershire Research Ethics Committee, and the study protocol was approved by the Research and Development Office of the University Hospitals of Leicester National Health Service Trust. The Primary Study group was identified by screening the Cardiology Department's audit databases for patients with a history of ICM who had undergone an electrophysiology study (EPS) with programmed electrical stimulation between January 1, 2005, and July 31, 2009, as part of clinical risk stratification for ICD implantation (in accordance with UK guidelines) and who had also had a CMR scan within 6 months of their EPS.^12^ This identified 43 patients. EPS recordings were unavailable for 9 patients, and 4 more patients were excluded because only 6-lead surface ECGs had been recorded. Of the 30 patients for whom EPS data were available, 3 were excluded from analysis because of insufficient data (noncaptured beats), and 1 could not be analyzed because the drive cycle length was changed midway through the protocol. CMR data were then sought for the remaining 26 patients. Late gadolinium-enhanced CMR images were not acquired for 3 patients because of difficulties with gating (n=1) and breath-holding (n=2), and 4 patients could not be analyzed because of an incompatibility between the acquisition and peri-infarct zone (PIZ) analysis software. Late gadolinium-enhanced CMR images were available for 19 of 26 patients.

A 30-patient Control group was selected from patients who had had an EPS for supraventricular tachycardia in 2010. Case matching was performed where possible on the basis of age and sex. Controls were excluded before data analysis if they had any of the following: insufficient surface ECG data recorded, an abnormal echocardiogram, atrial fibrillation, or family history of SCD or diabetes mellitus (because of potential for associated autonomic dysfunction and silent ischemic heart disease). During data analysis, 1 Control subject was excluded because it was apparent that the quadripolar catheter had moved from the right ventricular apex during the study. All Control subjects had normal left ventricular function as judged by visual assessment of 2D transthoracic echoes.

The Replication ICM group was selected in the same way as the Primary Study group, except that only patients who had ICD implantation were selected and a CMR scan was not required; this identified 40 patients.

### Electrophysiological Study

EPS were performed as per the standard departmental protocol, which did not change for the duration of the study. Fasting subjects were studied with minimal sedation and with antiarrhythmic drug cessation 4 to 5 half-lives before the procedure. Data were recorded with a 6F quadripolar catheter advanced transvenously to the right ventricular apex; other catheters were positioned as appropriate to the clinical procedure. Standard 12-lead ECGs were recorded with LabSystem Pro (Bard Electrophysiology, Lowell, MA) at a 1-kHz sampling rate, with a low-pass filter set to 50 Hz and high-pass filter set to 0.01 Hz. The study protocol used an 8- or 10-beat train at drive cycle lengths of 600 ms and 400 ms. A single-extrastimulus protocol was followed with decrements of 20 ms. If breakthrough beats were seen in the drive train, the drive cycle length was reduced. The S1–S2 coupling interval is the period between the last beat of the drive train and the first extrastimulus, and the R2I2 was derived from measurements taken from the last two S1 beats and the S2 beat. Programmed electrical stimulation was performed in the Primary Study and Replication cohorts. A modified Wellens protocol was used: 2 drive trains, drive cycle lengths 600 ms and 400 ms, at the right ventricular apex; and 1 drive train, drive cycle length 400 ms, at the right ventricular outflow tract; coupled with ≤3 extrastimuli with each drive train.^13^ Monomorphic ventricular tachycardia of duration >30 seconds or associated with hemodynamic compromise was recorded as positive; the test was otherwise recorded as negative.

### Analysis of the R2I2

The surface ECGs were exported at 16-bit digital resolution for analysis in custom software written in MATLAB (Mathworks, Natick, MA) by W.B.N. The timing of the QRS onset and T-wave peak (T_peak_) were analyzed automatically, and all data points were verified manually by W.B.N., a senior electrophysiology research fellow. The T_peak_ was chosen in preference to the end of the T wave (T_end_) because of the known difficulties in measuring the T_end_.^14^ The R2I2 is derived from the QRS onset and T_peak_ measurements. Intraoperator and interoperator reproducibility assessment was performed independently by 2 electrophysiology research fellows (W.B.N. and P.D.B.) using a representative sample of 242 paced ECG points from the dataset. Mean intraoperator variability for measurement of the QRS onset and T_peak_ was 5.0 ms (standard deviation 7.6 ms) versus interoperator 6.0 ms (standard deviation 7.9 ms).

**Figure 1.**
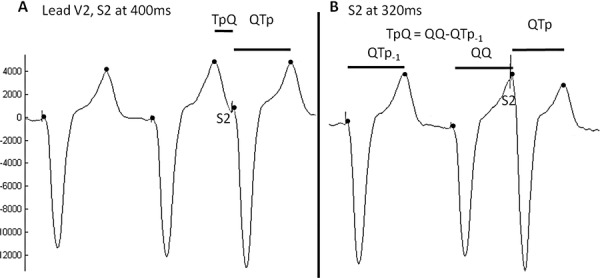
Technique for measurement of diastolic interval surrogate: T_peak_ to QRS onset (TpQ) and APD surrogate: QRS onset to T_peak_ (QTp). A. When an S2 arrives after the T_peak_ the TpQ and QTp are measured as shown on the left of the diagram. B. If the S2 occurs before the T_peak_ the TpQ is effectively negative. In this case it is measured by subtracting the QTp_-1_ interval (QTp for the last drive cycle beat) from the QQ, in the example above this would give a TpQ close to zero.

Data points were censored according to predetermined rules: (1) breakthrough beat occurring after beat 6 of the drive train (146 of 1114 drive train beats censored), and (2) point indeterminate because of artifact, baseline wander, or unclear morphology (536 of 11 758 points). For each S1–S2 coupling interval, the diastolic interval was taken as the period from T_peak_ on the last beat of the drive train to the S2 QRS onset (TpQ), as shown in Figure 1; note the possibility for negative T_peak_ to QRS onset interval as measured in this way. The body surface surrogate for the APD was taken as the period from S2 QRS onset to the S2 T_peak_ (QTp). These body surface surrogates were measured for each S2 performed at the right ventricular apex. Where possible, drive trains with drive cycle lengths of 600 ms were used, but if they were not present or were unusable because of breakthrough beats, an alternative drive cycle length was selected.

**Figure 2.**
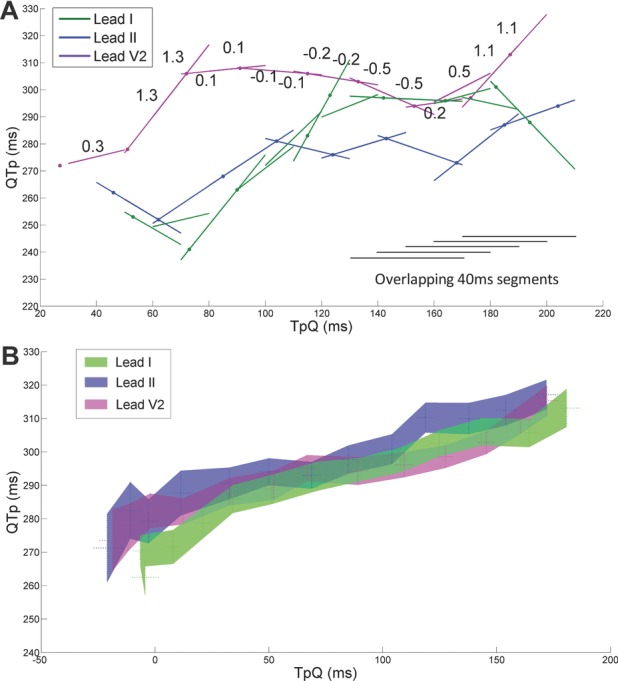
Explanation of the R2I2 calculation and demonstration of the body surface restitution relation. A. Plot of QRS onset to T_peak_ (QTp) against T_peak_ to QRS onset (TpQ) for representative ECG leads: I (lateral), II (inferior) and V2 (anterior) to explain the Regional Restitution Instability Index (R2I2) calculation in a typical study patient. For each lead, the QTp/TpQ gradient (least-squares regression) was calculated over a 40 ms segment of TpQ range. This segment was then scanned over the range of TpQ with available data to produce gradients at 10-ms intervals (example gradients are shown for lead V2). The difference of the gradient from the mean gradient in each 40 ms segment was calculated. The standard deviation of these values was taken as a measure of APD restitution heterogeneity in each lead. The mean of this was then taken as the R2I2. B. Mean data points for the individual ECG leads from the combined ICM cohorts were calculated and an area graph of the standard error of the mean has been plotted for the same representative leads as in A.

The focus of the study was on regional electrical heterogeneity assessed by APD restitution gradient. For each lead of the surface ECG, the QTp was plotted as a function of TpQ, and gradients were fitted by using 40-ms overlapping least-squares linear segments as described previously by Taggart et al ^15^ (Figure 2). For each lead, in each 40-ms segment, the difference of the gradient from the mean gradient in that 40-ms segment was calculated. The standard deviation of these values was taken as a measure of APD heterogeneity in each lead. The mean of this was then taken as the R2I2 (no units). A small number of nonphysiologically steep gradients result from points that have near or identical TpQ (measured to the nearest millisecond). To avoid skewing of the data, gradients exceeding ±10 were censored (1.5% of gradients).

### Measurement of T_peak_-to-T_end_ Interval and QT Dispersion

The exported ECGs also were used to measure nonpaced T_peak_-to-T_end_ (TpTe) intervals and QT dispersion. As described by Panikkath et al,^16^ TpTe was measured in lead V_5_, or if V_5_ was not suitable because of noise or low amplitude, V_4_ or V_6_ was used (in that order). T_end_ was taken to be the intersection of the tangent to the down slope of the T wave and the isoelectric line. If a U wave followed the T wave, the T-wave offset was measured as the nadir between the T and U waves. The QT interval was measured from the earliest onset of the QRS complex to the T_end_. The QT dispersion for each ECG recording was defined as the difference between the longest and shortest measured QT interval.^17^

### Late Gadolinium-Enhanced CMR Imaging Protocol

The patients with ICM in the Primary Study cohort underwent late gadolinium-enhanced CMR, as per departmental protocol, within a median of 35 (inter-quartile range, 105) days of their EPS. Comprehensive CMR imaging was performed via a 1.5-T scanner (Magnetom Avanto, Siemens) with ECG triggering and a 6-channel phased-array cardiac coil. After scout imaging, true fast imaging with steady-state precession (TrueFISP) cine images were acquired in 4-, 3-, and 2-chamber views, and a series of short-axis slices were obtained covering the left ventricle from base to apex, with 1 slice every 10 mm.^18^ A gadolinium-based contrast agent (0.1 to 0.2 mmol/kg) was administered intravenously as a bolus, and late gadolinium-enhanced images were obtained ≈10 minutes later with the use of an inversion-recovery, segmented gradient echo sequence.^19^,^20^

### CMR Analysis

**Figure 3.**
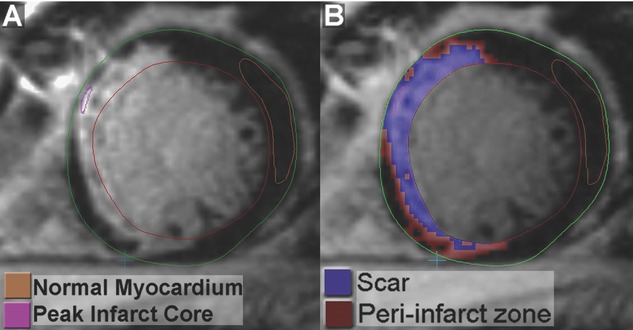
Endocardial and epicardial borders are drawn; then a large representative area of “normal myocardium” and a small area of “peak scar” are selected as shown in A. Software analysis identifies all voxels with signal intensity >2 standard deviations above “normal myocardium” mean intensity and voxels with signal intensity >50% of the “peak scar” are subtracted from this to obtain the PIZ. Identified voxels that are not in the region of an infarct are discarded.

All analysis was performed offline and blinded to patient details with commercially available software. Volumetric analysis was performed by manual tracing of endocardial and epicardial contours; left ventricular end-diastolic volume, end-systolic volume, stroke volume, left ventricular ejection fraction, and left ventricular end-diastolic mass were calculated. Late gadolinium-enhanced images were analyzed for scar and PIZ mass via a modification of the Schmidt et al^11^ technique. All voxels with signal intensity >50% of peak infarct core were recorded as scar. PIZ was defined as all pixels in the region of the myocardial infarction with signal intensity >2 standard deviations above mean intensity in an area of normal myocardium and <50% of the peak intensity (Figure 3). CMR volumes and mass were indexed to height. Scar size is presented as percent of left ventricular mass and PIZ as mass in grams, percent of left ventricular mass, and percent of infarct size.

### Statistical Analysis

Parametric data are expressed as mean ± standard error of the mean and were analyzed with the Student *t* test; nonparametric data are expressed as median [interquartile range; IQR] and were analyzed with the Mann-Whitney *U* test. Proportions were analyzed with a 2-sided Fisher exact test. A receiver-operator characteristic curve using the R2I2 was constructed in the Primary Study cohort for identification of the optimal R2I2 cutoff and for comparison in the Replication cohort. A Kaplan-Meier survival curve was drawn with the use of this cutoff value, combining the Primary Study and Replication groups; comparison of cumulative end points was based on logarithmic transformations. Survival was recorded as time to first end point or the end of follow-up. Pearson rank correlation was used to look for correlation between the R2I2 and PIZ, as well as between age and R2I2 within the Control, Primary Study, and Replication groups and between R2I2 and TpTe in the combined ICM cohorts. Spearman's rank correlation was used to look for correlation between R2I2 and QT dispersion in the combined ICM cohorts. A single Cox proportional-hazards model was used to look for independence of the R2I2, programmed electrical stimulation result, left ventricular ejection fraction, and QRS duration in the Primary Study group. *P*<0.05 was considered statistically significant. All analyses were performed with STATA (StataCorp LP, College Station, TX).

## Results

**Figure 4.**
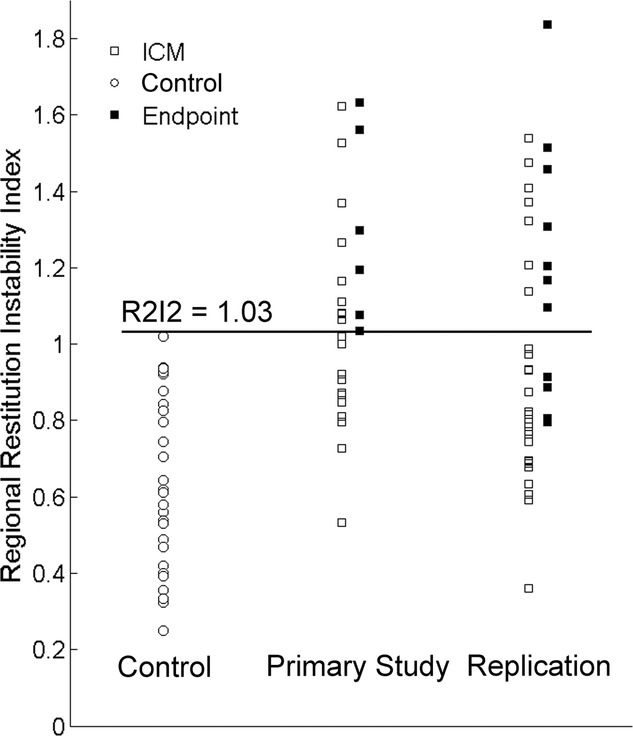
Scatter plot for Regional Restitution Instability Index (R2I2) in Control, Primary Study and Replication groups, the line indicates the value chosen to separate a positive and negative R2I2 result and filled squares identify patients who reached the endpoints of VA/death (Primary Study group) or VA (Replication group) during follow-up.

The clinical characteristics and relevant ECG characteristics, including mean R212 values, for the 26 Primary Study group patients, 29 Control subjects, and 40 Replication group patients are summarized in Table 1. Given that the Controls underwent an EPS for a different indication (supraventricular tachycardia), there were significant differences in their clinical characteristics and left ventricular ejection fractions compared to the 2 other groups. Individual-level R2I2 values for subjects of all 3 groups are shown in Figure 4. R2I2 was significantly greater in the Primary Study group than in the Controls (1.09±0.05 versus 0.63±0.04, *P*<0.001). No correlation was seen between age or sex and the R2I2 within the Primary Study, Control, or Replication groups.

**Table 1.** Main characteristics of the Primary Study, Control and Replication Groups

Variable	Primary Study Group (n=26)	Controls (n=29)	*P*	Replication Group (n=40)
Age, y	66.2±1.9	45.3±2.5	<0.001	66.2±1.5
Sex, % male	96	59	0.001	88
QRSD, ms	107±3.8	104±3.7	0.63	120±4.9
LVEF, %	29±2.7	64±1.0	<0.001	30–35%*
R2I2	1.09±0.06	0.63±0.04	<0.001	0.99±0.05

Parametric data are expressed as mean±standard deviation. (Abbreviations: QRSD QRS duration, LVEF left ventricular ejection fraction, R2I2 Regional Restitution Instability Index).

* The Replication group LVEF measurements were obtained from clinical echocardiogram reports that specify a LVEF range.

Median follow-up duration in the Primary Study group was 23 [IQR: 18] months, during which 6 patients reached the end point of VA or death: 3 VA and 4 deaths (1 patient had successful ICD therapy for VA and subsequently died). After programmed electrical stimulation, 12 of 26 Primary Study group patients had ICDs implanted, and 2 deaths occurred in 4 patients who did not have ICDs. Characteristics of the Primary Study group patients partitioned on the basis of the primary end point are shown in Table 2. Patients who reached an end point had significantly higher mean R2I2 than those who did not (1.30±0.10 versus 1.03±0.06, *P*=0.037). Age and programmed electrical stimulation result also trended toward an association with VA or death but were not correlated with R2I2. Cox multivariate analysis, including the R2I2, programmed electrical stimulation result, left ventricular ejection fraction, and QRS duration, showed that the R2I2 was an independent predictor of VA or death (*P*=0.029).

**Table 2.** Baseline characteristics of the Primary Study Group Split by Endpoint

Variable	No VA/Death (n=20)	VA/Death (n=6)	*P*
Age, y	64.5±2.2	71.8±3.5	0.120
Sex, % male	95	100	…
QRSD, ms	107±4.7	108±6.3	0.889
LVEF, %	30.5±3.3	25.3±3.0	0.414
PES result (positive/total)	7/20	4/6	0.348
R2I2	1.03±0.06	1.30±0.10	0.037
EDV index, mL/cm	1.43±0.12	1.44±0.23	0.958
SV index, mL/cm	0.37±0.04	0.39±0.06	0.790
Mass index, g/cm	0.80±0.04	0.72±0.04	0.342
Height, cm	170±1.7	173±2.2	0.383
Follow-up, mo	24[17]	19 [12]	0.273
PIZ%*	7.4[8.0]	15.6[4.6]	0.016
PIZ mass, g*	7.4[7.9]	20.1 [7.5]	0.033
PIZ mass/scar mass%*	66[64]	72[26]	0.643
Scar% left ventricular mass*	8.7[13.5]	22.0[3.8]	0.042

Parametric data are expressed as mean±standard deviation; nonparametric data as median [inter-quartile range]. (Abbreviations: QRSD QRS duration, LVEF left ventricular ejection fraction, PES programmed electrical stimulation, PIZ peri-infarct zone, R2I2 Regional Restitution Instability Index, EDV left ventricular end-diastolic volume, SV stroke volume).

* CMR PIZ data were available for 19 of 26 patients.

**Figure 5.**
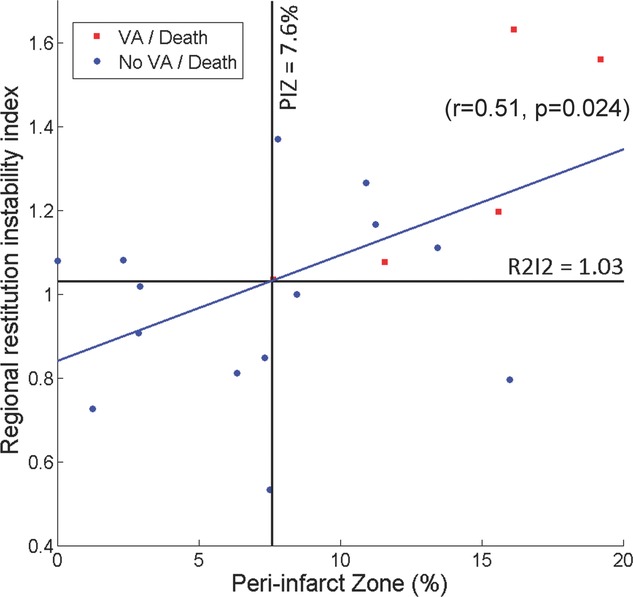
Plot of Regional Restitution Instability Index (R2I2) against peri-infarct zone (PIZ) in each of the 19 Primary Study group patients for whom paired data were available. Lines are drawn at the optimal cut-off values for both parameters. A least-squares regression line demonstrates significant correlation (r=0.51, *P*=0.024).

**Figure 6.**
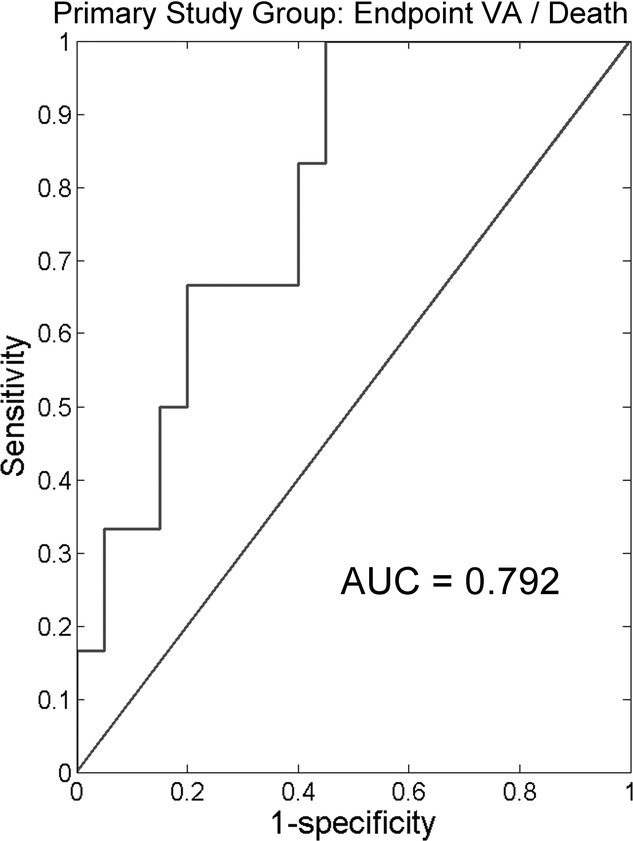
Receiver operating characteristic curve for Regional Restitution Instability Index (R2I2) in Primary Study group: VA/death versus VA-free survival.

The percentage of CMR PIZ was significantly associated with VA or death (15.6% [IQR: 4.6%] versus 7.4% [IQR: 8.0%], *P*=0.016) and exhibited significant correlation with the R2I2 (r=0.51, *P*=0.024; Figure 5). Other CMR parameters such as scar percentage also were significantly associated with VA or death (22.0% [IQR: 3.8%] versus 8.7% [IQR: 13.5%], *P*=0.042; Table 2). A receiver-operating characteristic analysis of the Primary Study group showed that an R2I2 cutoff of 1.03 provided the greatest discrimination between those who had a primary end point and those who did not have an event during follow-up (area under curve of 0.792; Figure 6). Interestingly, none of the Control patients had an R2I2 value >1.03 (Figure 4).

The Replication group did not differ significantly from the Primary Study group in the main clinical characteristics (Table 1). The Replication cohort subjects were selected on the basis of ICD implantation; therefore, the end point of VA was chosen. Median follow-up duration in the Replication cohort was 40 [IQR: 22] months, during which 11 patients reached the primary end point of VA and 7 patients died. The Replication cohort replicated the findings of the Primary Study group: Patients reaching the end point of VA had a significantly higher mean R2I2 (1.18±0.10 versus 0.92±0.05, *P*=0.019). An R2I2 cutoff value of 1.03 identified 7 of 11 patients who had an end point; the receiver-operating characteristic area under curve was 0.740.

**Figure 7.**
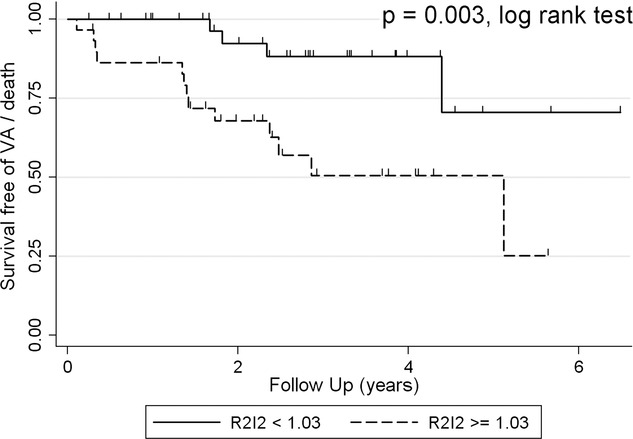
Kaplan-Meier survival curve for combined Primary Study and Replication groups showing a significantly higher rate of VA in a “high-risk” group with Regional Restitution Instability Index (R2I2)>=1.03 compared with the “low risk” group with R2I2<1.03 (*P*=0.003, log rank test).

In a combined analysis of the ICM subjects from the Primary Study and Replication groups, an R2I2 ≥1.03 identified subjects with a significantly higher risk of VA or death (43%; 13 of 30) compared with those with an R2I2 <1.03 (11%; 4 of 36) (*P*=0.004, Fisher exact). A survival curve of the combined ICM groups partitioned by an R2I2 value of 1.03 is shown in Figure 7; a highly significant difference (*P*=0.003, log rank) was observed. Side-by-side analysis of the R2I2 against TpTe and QT dispersion was performed for the combined ICM cohorts. In comparison of ICM patients experiencing VA or death versus ICM patients not reaching an end point, neither TpTe (88.8 [IQR: 32.7] versus 77.2 [IQR: 34.5], *P*=0.644) nor QT dispersion (66.3±32.8 versus 60.0±25.9, *P*=0.428) was associated with end points. There was also no correlation between R2I2 and TpTe (r=−0.004, *P*=0.978) or between R2I2 and QT dispersion (r=−0.207, *P*=0.096).

## Discussion

In this article, we report the development and characterization of R2I2 as a potential prognostic marker in patients with ICM at risk of SCD. Despite considerable effort, no definitive surface ECG marker of SCD risk has emerged to date. For example, microvolt T-wave alternans showed initial promise, but in the 490-patient substudy of SCD-HeFT, no significant difference in event-free survival was found between microvolt T-wave alternans–positive and –negative patients (hazard ratio 1.24, 95% confidence interval 0.60 to 2.59, *P*=0.56).^21^ Another technique is the signal-averaged ECG, which combines a series of QRS complexes to detect ventricular late potentials that are thought to correlate with VA risk. However, in CABG Patch, a 900-patient ICD prophylaxis trial, the signal-averaged ECG was unhelpful in identifying a high-risk group of patients.^22^ R2I2 is a novel approach, founded on improved basic science understanding of VA,^6^,^23^ that has the potential to be further refined and offer clinical utility.

In the Primary Study group, the R2I2 was significantly higher in patients with ICM who subsequently had a VA or died. This result was replicated in a Replication cohort of patients with ICM and the predefined end point of VA. The R2I2 electrical measure of risk showed a significant, moderate correlation with an anatomic measure of arrhythmic substrate, the extent of PIZ. Importantly, R2I2 was independent of programmed electrical stimulation result, left ventricular ejection fraction, and QRS duration, which suggests that it may add value to existing markers of risk of VA or death.

Simulation studies of ventricular fibrillation suggest that it is initiated by breaking of the depolarization wave into multiple wavelets that spread chaotically.^24^ Wavelets are extinguished if they collide with each other or are blocked by refractory tissue. Hence, according to this paradigm, ventricular fibrillation is maintained by the constant genesis of new wavelets. The electrical restitution properties of the heart have been linked to increased susceptibility to wavelet breakdown and generation of ventricular fibrillation. The relationship of the APD and conduction velocity of a certain beat to the diastolic interval from the previous beat has been shown both mathematically to be important and biologically to support a link with arrhythmogenesis.^6,25^ Regional heterogeneity of APD restitution adds to this electrical instability.^23^ Chronic myocardial infarction creates a complex milieu of heterogeneities in these electrical restitution properties. First, there is an underlying normal variation in APD restitution that is seen from base to apex, epicardium to endocardium, and left to right ventricle. Heterogeneous behavior of APD restitution in different regions of the heart has been demonstrated with intracardiac catheters and also with an epicardial sock of electrodes in patients undergoing cardiac surgery, with significant differences seen between patients with ischemic heart disease and aortic valve disease.^23,26,27^ Second, there is the anatomic heterogeneity related to interdigitation of infarcted and viable tissue and the effects of fibrosis on myofibril disarray and in particular conduction velocity restitution.^28–30^ Finally, there are the many sequelae of ischemia and myocardial insufficiency—eg, on APD and conduction velocity restitution curves and on the secretion of nerve growth factor leading to cardiac nerve sprouting and sympathetic hyperinnervation.^30,31^

**Figure 8.**
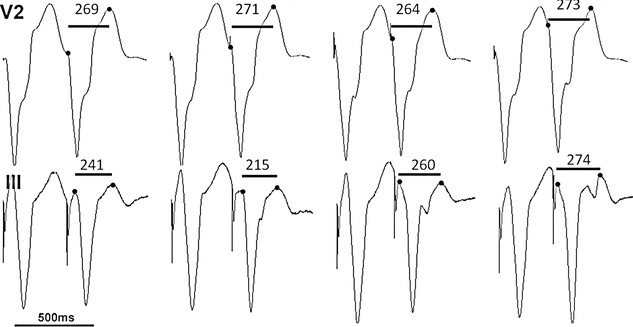
Diagram shows the last beat of the drive train and the S1 S2 coupling interval at 400, 380, 360 and 340 ms for leads V2 and III. Demonstration of regional heterogeneity in repolarization: little change is seen in V2 and the QRS onset to T_peak_ (QTp) is stable, while lead III is seen to fragment with 2 peaks and variable QTp. This Primary Study group patient had an R2I2 of 1.63 and had VA during follow-up.

A high-resolution 12-lead ECG describes the summation of myocyte electrical activity along specific vectors. Cardiac depolarization flows in a complex 3-dimensional wave that is influenced by static factors, such as myocardial scar, and dynamic factors that exert their influence through APD and conduction velocity restitution. The diastolic interval, which itself has a spatial gradient, affects the excitability of individual myocytes, regional conduction velocity, and also repolarization. These effects alter the depolarization wavefront, the consequence of this being dependent on whether the resulting wavelets propagate or are extinguished. In turn these effects will create spatial dispersion of repolarization and potentially discordant alternans.^32,33^ The R2I2 attempts to use the 12-lead ECG to measure regional manifestations of this irreducibly complex cardiac choreography. Figure 8 shows an example of regional differences in repolarization developing as the S1–S2 coupling interval shortens in a Primary Study group patient who went on to develop VA; these changes cause substantial heterogeneity in the restitution gradient and a high R2I2 (1.63).

The R2I2 uses T_peak_ as a surrogate for end repolarization rather than the more typically used T_end_ because detection of the former is more accurate than the latter. Antzelevitch et al1999 suggest that T_peak_ correlates with end-repolarization of epicardial cells, with the difference between T_peak_ and T_end_ (TpTe) reflecting transmural heterogeneity.^34^ Other authors consider the TpTe to reflect total left ventricular dispersion of repolarization.^35^ It may be that the QTp interval is as reflective of the intracardiac APD as the QT end interval, but the measures correspond to different components of the signal. Sundqvist et al^36^ investigated the relationship between QTp and QTe during exercise in healthy subjects; they found that the QTp and QTe decrease in parallel with increasing heart rate and that the majority of the decrease is in the QTp rather than the TpTe.^36^ Haapalahti et al^37,38^ have constructed QTp / heart rate slopes in control subjects and found them to be no different from QTe / heart rate slopes; they went on to find QTp autonomic responses in long-QT syndrome type 1 carriers to be more impaired than QTe responses. Important further work is needed to clarify the relationship between the R2I2 and intracardiac heterogeneity of restitution. Clinical studies by Panikkath et al^16^ and others have shown the TpTe interval to be a potentially useful predictor of SCD, and we will be actively assessing this measure and the QTe interval in future work.

For the electrophysiologist, the recent interest of the CMR community in assessing arrhythmia vulnerability through measurement of the PIZ is conceptually attractive. Although this approach is still at an early stage, it has been shown in a study of 144 post–myocardial infarction patients to be independent of left ventricular ejection fraction in its association with all-cause death (*P*=0.005), and it also has been found that PIZ predicted a positive programmed electrical stimulation test in 47 patients (*P*=0.015).^11^ Importantly, late gadolinium-enhanced CMR anatomic data have been shown to correlate with intracardiac CARTO (Biosense Webster) voltage data, histology, and cellular biochemical pathology.^39–41^ Limitations of PIZ quantification include spatial resolution of CMR (typically >1 mm), the range of methods used to delineate it, the different ways in which measured PIZ is expressed, and the tendency of PIZ research to use programmed electrical stimulation result as an end point.^10,11,39^ In the present study, we found a statistically significant correlation between PIZ and the R2I2 that offers further support for our hypothesis that R2I2 is an appropriate measure of electrical heterogeneity and of SCD risk.

The Replication cohort provided independent support for the efficacy of the R2I2 in predicting VA risk. Although this is encouraging, further work is needed to identify the potential role of the R2I2 in ICD risk stratification, especially in prospective studies. It would be interesting to perform side-by-side analysis of the R2I2 with other ECG markers of SCD, such as QT variability and T-wave alternans. This being a retrospective study, analysis of comparators was limited, but it is of note that there is no correlation between QT dispersion, a conceptually flawed measure of repolarization heterogeneity, and the R2I2.^42^ There recently has been interest in use of TpTe measurements, with Panikkath et al^16^ presenting encouraging data. In the present study, which represents a comparatively small cohort, TpTe values are higher in patients experiencing VA or death, but the result does not approach significance. The R2I2 does not correlate with the TpTe; the indices are substantially different, with R2I2 using TpQ and QTp to measure restitution gradient heterogeneity across the 12-lead ECG and with TpTe measuring repolarization heterogeneity in a single lead. However, the TpTe component of repolarization is potentially important, and as previously mentioned, we will be investigating TpTe and QTe in future work. There is scope for refining R2I2 and improving its accuracy. For example, decreasing the S1–S2 coupling interval in 10-ms steps from 300 ms could provide more detail in the steep portion of the curve. Work also is needed to validate the R2I2 against intracardiac data. Finally, whether determination of R2I2 could be undertaken entirely noninvasively by using exercise or chronotropic medication to induce a range of heart rates needs to be tested.

### Limitations

This is a small retrospective study and has several limitations. The R2I2 in its current form is invasive, requiring intracardiac pacing to create a spectrum of diastolic intervals. Only 19 of 26 patients' CMR scans were analyzable. If the R2I2 data had been collected prospectively, we would have used a longer drive train and repeated drive trains that had breakthrough beats, in addition to performing a dynamic restitution protocol. Multivariate analysis was limited by the small numbers.

### Conclusions

This pilot study suggests that the R2I2 is capable of extracting information on regional restitution heterogeneity and that this is increased in patients with ICM and is associated with VA or death. Further work is needed to refine the technique, as well as to explore the correlation between the R2I2 and intracardiac APD and conduction velocity restitution and their clinical significance in patients with ICM.
